# Using design strategies from microfluidic device patents to support idea generation

**DOI:** 10.1007/s10404-018-2089-6

**Published:** 2018-06-14

**Authors:** Jin Woo Lee, Shanna R. Daly, Aileen Y. Huang-Saad, Colleen M. Seifert, Jacob Lutz

**Affiliations:** 1 Department of Mechanical Engineering, University of Michigan, 2479 G.G. Brown, 2350 Hayward Street, Ann Arbor, MI 48109 USA; 2 Department of Mechanical Engineering, University of Michigan, 3316 G.G. Brown, 2350 Hayward Street, Ann Arbor, MI 48109 USA; 3 Department of Biomedical Engineering, University of Michigan, 2228 Lurie Biomedical Engineering, 1101 Beal Avenue, Ann Arbor, MI 48109 USA; 4 Department of Psychology, University of Michigan, 3042 East Hall, 530 Church Street, Ann Arbor, MI 48109 USA; 5 Department of Biomedical Engineering, University of Michigan, 2479 G.G. Brown, 2350 Hayward Street, Ann Arbor, MI 48109 USA

**Keywords:** Microfluidics, Design strategies, Creativity, Idea generation

## Abstract

Microfluidics has been an important method in providing answers to a wide variety of research questions in chemistry, biochemistry, and biology. Microfluidic designers benefit from instructional textbooks describing foundational principles and practices in developing microfluidic devices; however, these texts do not offer guidance about how to generate design concepts for microfluidic devices. Research on design in related fields, such as mechanical engineering, documents the difficulties engineers face when attempting to generate novel ideas. For microfluidic device designers, support during idea generation may lead to greater exploration of potential innovations in design. To investigate successful idea generation in microfluidics, we analyzed successful microfluidic US patents, selecting those with the key word “microfluidic” over a 2-year period. After analyzing the features and functions of 235 patents, we identified 36 distinct design strategies in microfluidic devices. We document each strategy, and demonstrate their usefulness in a concept generation study of practitioners in microfluidic design. While some of the identified design strategies may be familiar to microfluidic designers, exposure to this large set of strategies helped participants generate more diverse, creative, and unique microfluidic design concepts, which are considered best practices in idea generation.

## Introduction

The development of engineering devices has led to many important discoveries in complex biological systems including synthetic biology (Noireaux and Libchaber 2004; Agresti et al. 2010; Caschera et al. 2016), mechanobiology (Yang et al. 2011; Polacheck et al. 2013), single-cell analysis (Wheeler et al. 2003; Brouzes et al. 2009; Lee et al. 2016), and tissue engineering (Yang et al. 2001; Ma et al. 2005). Microfluidic technology, in particular, has become a power tool for biologists and biochemists as microfluidic devices can miniaturize macroscopic systems to control cellular microenvironments and reduce reagent consumption (Whitesides 2006). As microfluidic practitioners solve problems in varied areas, including medical diagnostics (Tse et al. 2013), drug screening (Pihl et al. 2005; Dittrich and Manz 2006), and single molecule studies (Dittrich and Manz 2005), they are frequently navigating open-ended design problems. However, the field of microfluidic design lacks research-based strategies to support practitioners in developing concepts to solve these design problems. Additionally, while microfluidic textbooks provide fundamental understandings of physics behind fluid transport and fabrication methods, they do not often describe idea generation approaches to develop potential device concepts.

Idea generation, as a process, is part of what is referred to as the “front-end” of design (Zhang and Doll 2001). Understanding front-end processes is important because critical decisions such as setting new device features are determined during this stage. The front-end phase also has the largest potential for changes and improvements with the least effort because it focuses on conceptual rather than implemented designs (Cooper 1993; Verganti 1997). Particularly, idea generation has been shown to be a critical part of a front-end design process because innovation is often traced back to initial idea creation (Brophy 2001). By considering multiple concepts that are diverse and creative, designers can select desirable features for future development and testing. However, research has demonstrated that engineering designers often fail to consider multiple alternatives and become very focused on specific options early in the design process. Designers tend to fixate on their initial ideas and design specifications (Purcell and Gero 1996; Sio et al. 2015), resulting in the replication of existing solutions. This replication often results in reproducing design flaws, especially when designers have already seen possible solutions (Jansson and Smith 1991). In actuality, exciting new designs often depart from previous designs (Baxter 1995).

Several strategies have been developed in design fields to assist developers in generating multiple solutions, such as brainstorming (Osborn 1957), morphological analysis (Allen 1962), Design Heuristics (Yilmaz et al. 2016a), SCAMPER (Eberle 1995), and TRIZ (Altshuller 1997). Each of these strategies brings a unique approach to directing ideation. Brainstorming includes general guidelines such as propose many ideas, avoid evaluation of ideas, and build off of others’ ideas (Osborn 1957). SCAMPER provides general theme suggestions (substitute, combine, adapt, modify, put to other uses, eliminate and rearrange/reverse) for developing new designs by changing existing designs. TRIZ, developed from a study of patterns and strategies in patents, suggests particular strategies based on contradictions in current designs (Altshuller 1997). Design Heuristics, developed from studying award-winning products and expert engineers’ practices, assist product designers through a collection of strategies that can be applied individually or in combination to initiate or transform ideas (Yilmaz et al. 2016a).

TRIZ was developed to improve products, services, and systems. Design Heuristics were developed to be used in product design settings to create tangible inventions, but have been shown to be applicable to other design domains (Ostrowski et al. 2017; Lee et al. 2017). This is to be expected as some design strategies are considered domain general, meaning they can be generalized across multiple contexts (Daly et al. 2012a). Thus, there are likely strategies within these approaches that are transferable to support idea generation for microfluidic designers. However, some strategies in design are also considered to be domain specific (Blessing and Chakrabarti 2009), and there are likely ideation strategies unique to microfluidics.

The goals of this work are to identify strategies used specifically in the domain of microfluidic design using the TRIZ development approach of patent analysis, and to compare the resulting strategies to product designs in mechanical engineering, particularly analyzing the transferability of Design Heuristics. The degree of overlap will identify important ways in which microfluidic design differs from other types of mechanical engineering design. The specialized applications in microfluidics may give rise to alternative design strategies unique to this field. By considering design strategies in microfluidics, we will add to our understanding of the challenges within microfluidics design, as well as identifying strategies that may be helpful to the design of new innovations.

While microfluidic designers may be familiar with some design strategies from their prior experiences, past research indicates that using a set of strategies expands the set of concepts considered (Gadd 2011; Ilevbare et al. 2013). Particularly, professional engineers who have been working in a design context for an extended time can benefit from utilizing design strategies (Yilmaz et al. 2013). Professional engineers often work on the same products over many years, gaining experience and expertise in a given domain. An expert designer may already accumulate design strategies from experience, but even experts may not consciously access all relevant strategies during a design process. Thus, using an idea generation tool during design may stimulate original thinking and elaboration of ideas (Yilmaz et al. 2013). The explicit communication of a collection of microfluidic design strategies may serve to remind microfluidic designers of broader possibilities, and offer a systematic way of considering multiple and diverse solution options. In addition, the analysis of recent patents has the potential to identify previously undocumented, domain-specific design strategies for microfluidic devices.

We also investigated the utility of design strategies for microfluidic design. An empirical study with advanced graduate students in microfluidics demonstrates the application of the identified strategies to novel problems. Demonstrating their utility in this study will support the use of these strategies by both novice and expert practitioners in the microfluidics community.

### Uncovering design strategies

Idea generation is a complex cognitive process that many researchers have investigated (Dinar et al. 2015). By studying expert designers, researchers have uncovered that designers draw from knowledge developed from their experiences (Schunn et al. 2005), restructure design problems through transformation (Goel and Pirolli 1992), and connect experiences and retrieve memories (Cross 2003) as they generate ideas. By building experiences and developing expertise, designers learn to use domain-specific knowledge that can be translated into simple strategies to make decisions (Schunn et al. 2005).

To uncover strategies in microfluidic design, we modeled our research method after prior studies of idea generation techniques in product development, TRIZ and Design Heuristics. Altshuller’s TRIZ, a “theory of inventive problem solving,” was developed by comparing over 40,000 patents, and resulted in 40 principles for finding effective solutions (Altshuller 1997). The resulting method assists engineers in identifying and resolving problems in the implementation of designs by finding existing solutions and adapting them to the current problem. For example, in designing eye medications, liquid drops are easy to dispense in the proper quantity, but can drain into the cul de sac of the eye. Using TRIZ principle #35 Change physical or chemical parameters, a designer may change the liquid eye medication to a gel. In this manner, TRIZ principles capture patterns to resolve design tradeoffs and technical conflicts.

Altshuller’s patent analysis method identified repeated patterns of technical solution types, and Altshuller suggested that these patterns could be applied to solve new design problems (Altshuller 1997). TRIZ includes a large set of interrelated tools, such as flowcharts and tables mapping principles to a given problem; as a result, in-depth training is required. Yet, TRIZ has been successfully implemented in engineering classrooms; for example, in one study, undergraduate and graduate mechanical engineering students at two universities were trained to use the TRIZ Contradiction Matrix in two 50-min lectures and asked to redesign a traffic light (Hernandez et al. 2013). The results showed that TRIZ improved ideation effectiveness as measured by the quantity, novelty and variety of ideas (Shah et al. 2003). TRIZ has been adopted in mechanical design industries to aid in idea generation (Tsai et al. 2004; Cascini and Rissone 2004) for design problems such as a polymeric wheel and metal-seated ball valve. The TRIZ method shows that useful design principles can be identified from patents, and serve as a valuable tool in idea generation and problem solving. In the present study, we employed a similar method involving a qualitative analysis of patents in the domain of microfluidics as a means of identifying patterns in device designs.

Design Heuristics is an idea generation technique developed from triangulation across a series studies in the product design context (Yilmaz et al. 2016a). In one study, researchers analyzed the functionality, form, user-interaction and physical state of over 400 award-winning consumer products. Designs with the same apparent innovations were grouped, and their commonalities and abstract patterns were identified (Yilmaz et al. 2016b). Other studies included a longitudinal case study of a single designer creating over 200 product designs (Yilmaz and Seifert 2011), and protocol studies of students and practitioners as they worked on a novel product design task (Daly et al. 2012c). The Design Heuristics approach has also been empirically validated as a useful technique for generating designs (Kramer et al. 2015). Students in mechanical engineering have been shown to benefit from the explicit instruction in these strategies for product design, and their use leads to resulting concepts that were more creative, practical, and original (Kramer et al. 2014). In addition, practicing expert industrial engineers were found to benefit from the use of Design Heuristics as strategies for creating new designs for an existing product line (Yilmaz et al. 2013).

Design Heuristics leverage the notion of the psychology term “heuristic,” which refers to a cognitive strategy known as a “short cut,” an educated guess that comes from experience and lessons learned (Maier 2009). Cognitive heuristics are “best guesses” at possible solutions, and they do not always provide a satisfactory solution. Instead, heuristics can provide direction that reduces the search for a satisfactory solution (Koen 1985). Past research shows that experts leverage similar heuristics in problem solving, and use domain-specific heuristics in expert performance (Klein 1999). For example, fire fighters arriving on an emergency scene have to make decisions to initiate search and rescue and identify where to allocate resources. Expert fire fighters recognize which approach to take in a new problem setting based on their prior experiences with other events, which serve as heuristics guiding their decision-making processes (Klein 1993).

**Fig. 1:**
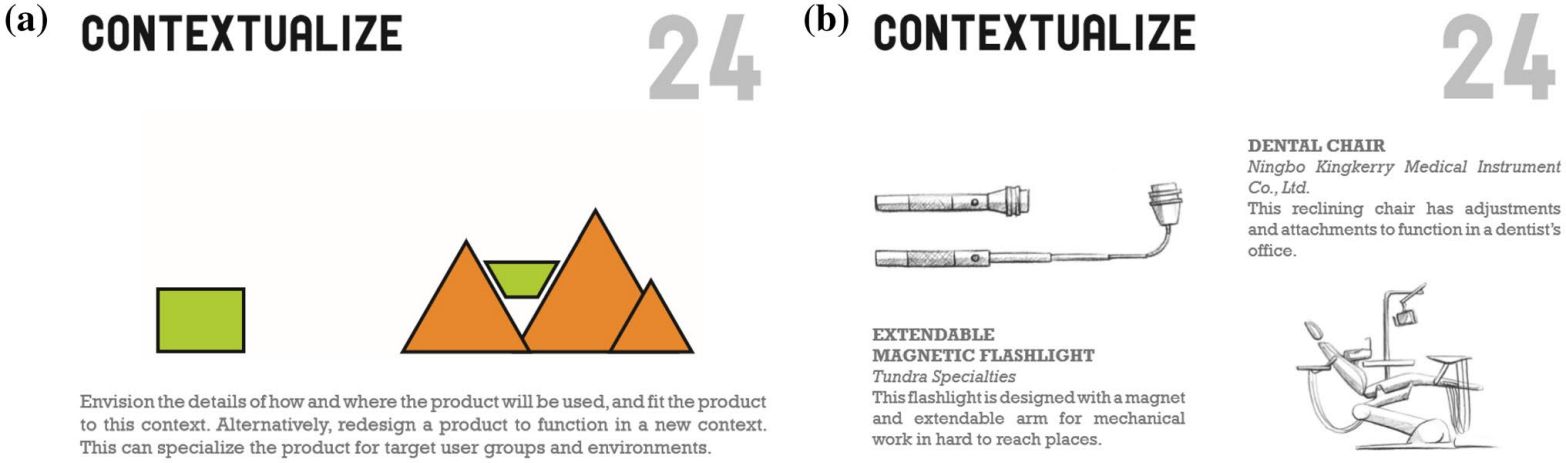
Design Heuristic card #24 (of 77), contextualize. **a** Front of card features a written description and graphic image; **b** back of card shows two example designs in which this Design Heuristic was evident

Design Heuristics are cognitive strategies intended to provide short-cuts to uncover potential design solutions. They were defined at a level of generality that can apply across multiple products, but specific to be observable in a single design (Yilmaz and Seifert 2011). For product design, a set of 77 Design Heuristics was identified (Yilmaz et al. 2016a) (Table 3 in Appendix 2). An example of Design Heuristics is contextualize, which prompts the designer to consider how the product will be used in a specific context (Fig. 1). In designing a microfluidic device for low-resource settings, the designer may consider the differences and limitations of the setting, such as limited electricity, equipment, and healthcare workers. With this in mind, the designer may pursue a low-cost, hand-powered device usable with minimal training (Bhamla et al. 2017). By pushing designers to consider variations based on successful designs, Design Heuristics can help both novice and expert designers to broaden the variety of ideas they generate and consider more concepts (Daly et al. 2012b).

Microfluidic design has several differences from product design due to differing fluid phenomena occurring on the micro/nanoliter scale. Also, microfluidic devices have several applications in chemical analysis and molecular biology, which may require materials with bio-chemical compatibility. Because both product and microfluidic design aim to develop tangible deliverables, there are likely transferable elements across the two domains. Thus, there may be some overlap among Design Heuristics and the strategies of idea generation in microfluidics. In addition, the differences in microfluidics may result in some unique design strategies specific to microfluidics. To explore the possible role of design strategies in microfluidic design, we studied a large sample of novel designs available as successful patents in microfluidics. In this study, we aimed to systematically investigate the design strategies present in microfluidic devices. By examining and identifying design strategies in microfluidics, we aimed to support the development of tools to aid idea generation for microfluidic designers to guide the discovery of novel ideas that could result in innovative outcomes in the field.

## Research design and results

Two different studies were conducted in the development of microfluidic design strategies. (1) The primary study analyzed common design patterns from microfluidic patents to develop a list of design strategies. (2) We validated the utility of the design strategies by conducting an idea generation study with graduate students and postdoctoral researchers involved in microfluidic research.

### Study #1: patent analysis

#### Method

The purpose of our study was to uncover strategies in the design of microfluidic devices. The following research question guided our study:

- What strategies or commonalities are evident in microfluidic device designs?

We chose to use microfluidic patents as our data source since patents focus on the description of design elements of microfluidic devices, compared to journal papers that often focus more on answering scientific questions. Analyzing patents may provide a variety of ideas in the microfluidic domain because patents represent inventions and designs that are novel, useful, and non-obvious. In addition, patents often contain work done in both academia and industry, which can make the identified strategies applicable in both domains.

##### Patent data collection

We collected patents with file dates between May 2014 and May 2016 in the United States Patent Office database using the key word, “microfluidic,” for a total of 508 patents in the sample. The patents were, then, screened for relevance to microfluidic design by excluding those that: (1) referenced microfluidics only as a possible platform for the method, and (2) only contained the word “microfluidic” in patent citations but not the device. From this screening process, 235 of the patents (46%) were included in the study.

##### Patent analysis

**Fig. 2:**
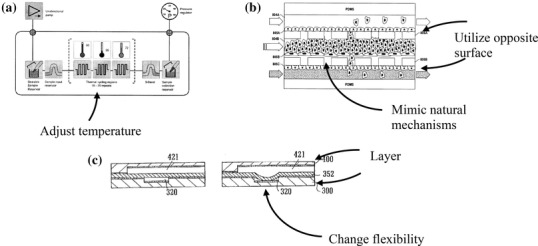
Examples of strategies identified from patents. **a** (McCormack et al. 2016) temperature-controlling element that suggested the strategy *adjust temperature*. **b** (Ingber and Huh 2016) An organ mimic device that suggested the strategies *utilize opposite surface* and *mimic natural mechanisms*. **c** (Jarvius and Melin 2016) A microfluidic device that suggested the strategies *layer* and *change flexibility*

For each patent, we examined the abstract, claims, basic summary, and images provided. We identified the intended function of the patent and its key features. Next, we considered the variations evident in the design for each function, and the claim for the novel design elements of the patent. We looked for characteristics that differentiated each patent. We, then, attempted to describe the design as a more general strategy that might be applicable to other designs. As an example, a patent labeled “fabrication and use of a microfluidics multitemperature flexible reaction device” (Fig. 2a) used temperature-controlling elements along its channels (McCormack et al. 2016), suggesting the strategy *adjust temperature*. Another example, “Organ mimic device with microchannels and methods of use and manufacturing thereof” (Fig. 2b) described a microfluidic device that would mimic the complex architecture of the lung alveoli (Ingber and Huh 2016), suggesting the strategy *mimic natural mechanisms*. Also, the surfaces of membranes were coated with cell adhesive molecules to support attachment of cells on both sides of the membrane, which demonstrated the strategy *utilize opposite surface*. “Microfluidic structure” (Fig. 2c) provided an example of a microfluidic device that was comprised of elastomer that can be deformed to control the fluid (Jarvius and Melin 2016), suggesting the strategy *change flexibility*. In addition, the device used multiple layers, such as the elastomer layer and bonding layer, to provide additional functionality, suggesting the strategy *layer*. By analyzing the functions of various patents, the goal was to identify design strategies that were present in multiple microfluidic designs.

We began the analysis with the existing set of Design Heuristics identified in product designs. If the strategy was consistent with one of the existing Design Heuristics, it was coded as a transferable strategy. If the strategy was not previously identified, it was listed as a potential new microfluidic design strategy. Two coders analyzed a subsample of 100 patents and coded for the presence of specific strategies, with each new strategy added to the coding set. Identification of new strategies occurred frequently in analyzing the first several patents, while only a few strategies were added towards the end of the subsample analysis, suggesting saturation.

At this point, some of the strategies with similar descriptions were either revised or combined by the two coders working together. Each coder independently coded and compared the results of 30 patents in the sample. One coder was an undergraduate student with experience in microfluidic design, and the other held an M.S. in engineering with past research in microfluidics. Their inter-rater reliability (Stemler 2004) was above 74% and any disagreements were resolved through discussion. Values greater than 70% are typically acceptable for inter-rater reliability (Osborne 2008; Gwet 2014). The remaining patents were analyzed by a single coder. Once the sample analysis was complete, we conducted a comparison of ten patents selected at random in each of the 4 years prior to the sample (2010–2013) to identify any new design strategies not yet identified. This new sample of 40 did not display any new strategies, suggesting that the sample size for the study was adequate for identifying a broad sample of microfluidic design strategies.

#### Results of the patent analysis

A collection of foundational microfluidic design strategies emerged from the analysis; 51 repeating design strategies were identified, as each strategy was observed in at least 2 of the 235 patents in the sample. Of these, 34 could be described by one of Design Heuristics (Table 3 in Appendix 2) and 17 were new strategies uncovered in the microfluidics patent data (Table 4 in Appendix 2). Within the 34 Design Heuristics identified, 19 were considered transferable (Table 5 in Appendix 2) and 15 were defined as “inherent strategies” (Table 6 in Appendix 2), to convey that these characteristics are qualities of most microfluidic devices. For example, the Design Heuristics, hollow out, was considered an inherent strategy because most microfluidic devices require cavities and hollow channels to allow fluid to flow. Another inherent strategy was expose interior; since many microfluidic devices require microscopy image processing and analysis, transparent materials are commonly used.

**Fig. 3:**
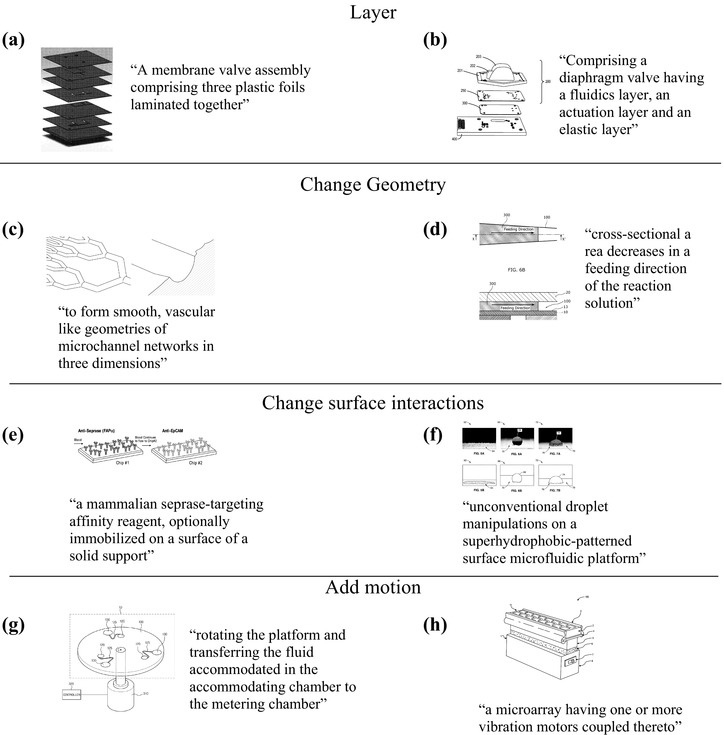
**a** (Sjolander 2016) and **b** (Vangbo 2015) example microfluidic designs for layer. **c** (Borenstein et al. 2016) and **d** (Tachibana et al. 2016) example devices for strategy change geometry. **e** (Martin et al. 2016) and **f** (Pan and Xing 2016) example designs for strategy change surface interactions. **g** (Lee 2015) and **h** (Bell et al. 2016) example devices for strategy add motion

The new design strategies and the transferable Design Heuristics strategies, but not the “inherent” strategies, are considered to be the working set of microfluidic design strategies. Examples of four microfluidic strategies and excerpts from patents that provided evidence are shown in Fig. 3. The strategy *layer* described devices that were built using layers of similar or different materials. Different layers provided varied functions; for example, a laminated microfluidic device included membrane valves (Fig. 3a), with each layer regulating the flow of a liquid sample in separate flow channels implemented in different layers (Sjolander 2016). Another device (Fig. 3b) had a fluidic layer, actuation layer, and elastic layer, and the elastic layer served as a barrier between the fluidic and actuation layers (Vangbo 2015).

Another strategy, *change geometry*, was evident in devices that would alter the typical or expected geometric form of the device or components while maintaining function. This can be helpful in redefining user interactions or suggesting new device functions. Developing microfluidic devices has often relied on microfabrication processes using lithography or etching techniques to pattern parts on a substrate. Photolithography involves coating, exposing, and developing photoresist in layers of rectangular fluid channels and shapes. One device (Fig. 3c) had rounded channels that differed from the typical rectangular channels (Borenstein et al. 2016), and this vascular-like geometry allowed better control of wall shear stress and flow conditions. Another device (Fig. 3d) used tapered channels to control the speed of the moving fluid (Tachibana et al. 2016). In a passive flow condition, due to the capillary action, the velocity of the fluid would decrease as the fluid advanced; by decreasing the cross-sectional area of a channel, the fluid can move at a constant velocity.

The strategy *change surface interactions* described how samples would interact with a device by changing hydrophobicity or allowing molecules to be captured, improving function and usability. In one device (Fig. 3e), antibodies were coated on the surface to capture circulating tumor cells, the source of cancer metastasis (Martin et al. 2016). Another device (Fig. 3f) demonstrated a super hydrophobic-patterned surface that repelled water to implement surface tension-driven flow without a pump (Pan and Xing 2016).

For the strategy *add motion*, evidence included devices that added movement or motion as part of the function. This can improve function or change user interaction; for example, one device (Fig. 3g) included a rotating platform for transferring fluid from one chamber to the next instead of a pump to transfer fluid (Lee 2015). Another device (Fig. 3h) incorporated a microarray of vibration motors to assist in mixing aqueous solutions (Bell et al. 2016). These examples illustrate the presence of design strategies identified in multiple microfluidic patents.

Table 1 presents the working collection of microfluidic design strategies, 19 transferable and 17 new design strategies, with example devices. Each strategy relates to specific features or characteristics of a device. These example devices are solely meant to illustrate the design strategies. How each strategy is displayed differs based on the design problem.

**Table 1:** 36 strategies identified in the analysis of microfluidic patents

Add modularity	Add motion	Add to existing product	Adjust functions for specific users	Adjust temperature	Allow user to customize
					
This device is made up of cubes that each has one function. Assembled together, they make a functional device (Bhargava et al. 2015)	The platform of this device rotates to improve fluid movement and separation (Lee 2015)	This device reads fluorescent signals from a microfluidic assay that has already completed its reaction(s) (Handique and Brahmasandra 2014)	This is a regulator of intra-ocular pressure (IOC) that is inserted into patients with glaucoma. The amount of vitreous humor that needs to be removed is specific to each patient (Gunn and Johnson 2014)	The picture depicts the process in a microfluidic thermal reactor cassette (McCormack et al. 2016)	A breadth sensor, which performs multiple functions using the mouthpieces in specific combinations to obtain the required results (Vrtis and Landini 2009)

Apply an existing mechanism in a new way	Attach independent functional components	Attach product to user	Automate	Change device and or sample lifetime	Change flexibility
					
This depicts separation of particles using ultrasound (Rose et al. 2016)	This device performs many different functions to isolate DNA in a single microfluidic device (Stone 2016)	A portable insulin pump that attaches to the user’s arm	This device provides an automatic readout once a sample is inserted (Whitesides et al. 2014)	This device can be stored and packaged with filled reservoirs for 6–12 months (Erkal et al. 2014)	This device has a flexible membrane that controls the fluid (Jovanovich et al. 2016)

Change geometry	Change surface interactions	Change volume to surface area ratio	Contextualize	Convert 2-D to 3-D	Create a multi-phase system
					
This device uses different surface shapes for cells to interact (Toner et al. 2015)	This device captures cells by coating the chamber with binding moieties (Jiang et al. 2016)	The change in surface area affects how much the sample interacts with the surface of the device (Haam et al. 2016)	A paper-based microfluidic device that detects pathogens. Due to its low-cost and easy setup, this device can be accessible to countries that lack the resources to detect pathogens (Jain et al. 2015)	This device mimics 3-D cell culture conditions (Toner et al. 2015)	The image above shows the process for Co(II) wet analysis using CFCP. This multi-phase flow consists of substances in both solid and liquid phase (Sato et al. 2003)

Embed electronics or electrodes	Impede flow	Incorporate environment	Incorporate filtration, separation, and/or sorting	Incorporate user input	Incubate
					
The image above shows external electrodes manipulating objects in microfluidic devices (Molho et al. 2016)	Micropillars are used to block particle movement, which impedes movement (Kopf-Sill 2016)	A retinal prosthesis device that uses a light-powered microactuator as a microfluidic pump (Saggere et al. 2009)	A particle sorting module that helps to filter the cells of interest from the sample (Johnson et al. 2015)	A portable glucose monitor that can analyze a blood sample for glucose levels, peak flow meter, etc., depending on the user’s input (Brown 2010)	The bottleneck shown allows more time for chemical reactions in the droplets to take place (Frenz et al. 2015)

Layer	Merge droplets	Mimic natural mechanisms	Run on passive flow	Run parallel operations	Simplify sample preparation
					
This device has three plastic layers, which can provide added functions (Sjolander 2016)	This device has two reservoirs, each with a different kind of droplet, and merges them into a channel (Kiani et al. 2015)	This device mimics osteocyte networks found in bone tissue (Lee et al. 2015)	This device creates an inherent pressure gradient, allowing for passive fluid flow (Pan and Xing 2016)	This device executes multiple operations at the same time (Ganesan 2015)	This device analyzes whole blood (courtesy of Abbott Point of Care Inc., NJ, USA)

Slide	Substitute way of achieving function	Use a disposable cartridge	Use colorimetric or fluorescent Imaging	Use magnets or a magnetic field	Utilize opposite surface
					
This device controls the flow by sliding (Davies and Dalton 2010)	This device is an implant glucose monitor and insulin regulator that does not require any external electricity supply, insulin reservoir, nor external pump (courtesy of Medtronic, MN, USA)	This picture shows a cartridge being inserted into a part of a larger microfluidic device (Dodd et al. 2015)	This is a microarray, a device used to analyze genes. The different-colored fluorophores that light up indicate the detection of different types of genes	This device uses magnetic beads to sort and manipulate droplets (Chang and Cheng 2016)	This device uses both surfaces to culture different types of cells (Ingber and Huh 2016)

### Study #2: validating the utility of microfluidic design strategies

#### Method

##### Empirical study using the microfluidic device design strategies

To explore the perceived value of the microfluidic device design strategies for microfluidic researchers and the types of designs generated using the strategies, we conducted an empirical study. In a single test session, participants with experience designing in the field of microfluidics were invited to use and apply the strategies to their own (or a provided) microfluidic device design problem. The goal of the empirical study was to identify the impact of design strategies in idea generation. In particular, we asked participants to assess the diversity, creativity and uniqueness of the ideas generated. The study was motivated by the following research question:

- Can these design strategies support idea generation in the microfluidic domain?

##### Study participants

An email was sent out via a listserve of microfluidic researchers (graduate students, postdoctoral fellows, and faculty) at a large Midwestern University to invite them to learn about a proposed tool in microfluidic device design and participate in using it. Fourteen participants completed the study, with nine males and five females (seven whites, one black, three Asians, three no responses). Eleven graduate students (4 second years, 4 third years, 2 fourth years, and 1 fifth year) and three post-doctoral researchers participated. The participants’ average age was 25.4 years, and they reported an average of 2.6 years of experience in microfluidics, most in academic research. Participants were active researchers who have been working in microfluidics, including two participants who had published in *Lab on a Chip*, and four more participants who had published in other microfluidic-related journals such as *ACS Nano* and *Advanced Materials*.

##### Study data collection and analysis

The session lasted for 80 min. Participants were encouraged to work on their own research problems, and ten participants did so. Four participants were given example problems, and chose one of the two example problem statements in Appendix 1. The sequence tasks in the study session included: (1) problem definition, (2) introduction on idea generation, (3) individual ideation, (4) design strategies lesson, (5) individual ideation with design strategies, and (6) reflection survey.

First, participants wrote a description of their problem (10 min). Next, participants were instructed for 5 min about best practices of idea generation in design, including principles such as generating multiple ideas and not evaluating ideas during generation. Then, participants were instructed to create four concepts to address their microfluidic design problem. They had 20 min to work independently using their own, natural methods to develop ideas. Participants recorded each idea by sketching each concept and writing a short description on a sheet of paper.

**Fig. 4:**
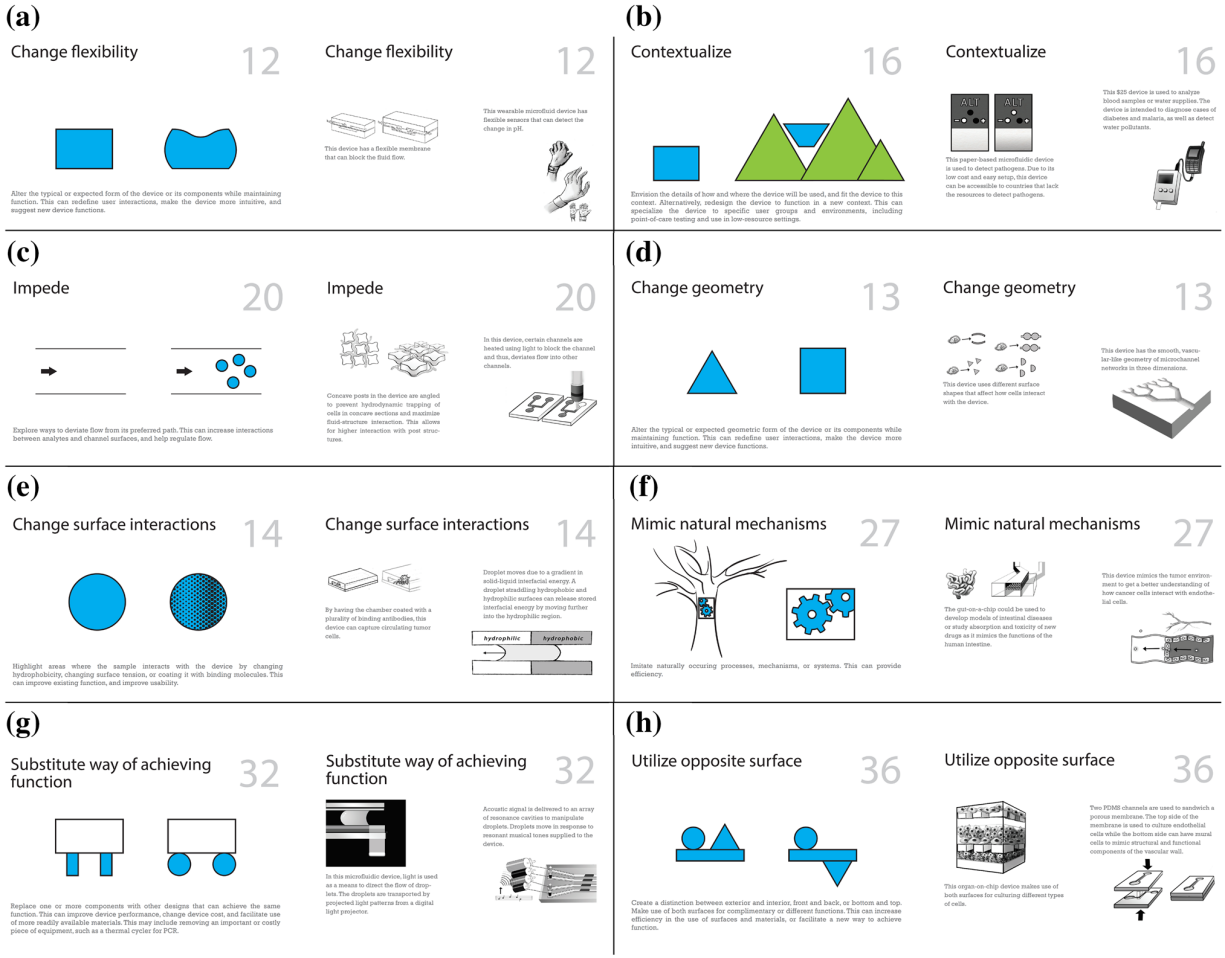
Eight design strategies used in experimental session. Each strategy was printed on a card. The front of each card has the title, abstract image and description. The back has two examples of microfluidic devices that incorporate that design strategy **a** change flexibility, **b** contextualize, **c** impede, **d** change geometry, **e** change surface interactions, **f** mimic natural mechanisms, **g** substitute way of achieving function, and **h** utilize opposite surface

Then, the facilitator provided a 10-min introduction to the microfluidic design strategies that emerged from the patent analysis (above). This included practice in applying two of the strategies with a new microfluidic device design problem (different from those in Appendix 1). To minimize any bias from the research team, we did not lead or prime participants to assume that using design strategies may help them generate better quality ideas. Instead, we focused on the potential usability of design strategies in generating ideas for their design problems. Next, participants generated up to four more concepts using a subset of eight of the microfluidic device design strategies. Each of the strategies were provided to the participants on a card, where the front of the card included a description of the strategy and a graphic image representing its use, and the back of the card provided two examples (Fig. 4). We considered this a manageable number of strategies to read and try to apply in the time allotted. Overall, 45 ideas were generated by participants when using their own approaches, and 40 ideas were generated with the microfluidic device design strategies. Analysis included documenting participants’ self-reported design strategy use, and we counted how many times each strategy was reported by the participants.

Finally, participants completed a short survey in which they assessed the concepts they generated, both using microfluidic device design strategies and without them (Appendix 2). Participants were asked to select one concept that was “most creative” out of up to eight ideas they had generated, with four from the first session and four from the strategy use session. They also rated the creativity, and uniqueness of each of their concepts, and the diversity of both sets. For ratings of individual concepts, participants were asked to choose only one idea for each quality (i.e., creativity, uniqueness), and for rating of the idea sets, participants could select only one set of ideas, rather than allowing participants to indicate that certain qualities may be equal between two ideas or two sets of ideas.

When participants rated their own ideas, we intentionally did not define the word “creativity.” While creativity has been defined in many ways in the research literature, broadly speaking, creativity constitutes something that is novel and useful within a field (Runco and Jaeger 2012). However, leaving creativity undefined in assessments of ideas is consistent with the way many creativity researchers measure the creativity of ideas as intentionally leaving creativity undefined does not impose a bias that may be inconsistent with the views on what is creative by experienced individuals in a field (Hennessey 1994; Baer et al. 2004). Experienced professionals with domain knowledge in a particular field have been shown to share creativity criteria (that is based on experience and intuition built by engaging in a field) and agree on what is considered creative (Amabile 1982). Since content knowledge is critical in judging creativity, and participants in this study were experienced in their domain, these participants were in the best position to evaluate the creativity of ideas.

After participants assessed their ideas, a chi-squared test of homogeneity based on frequencies was conducted to determine the distribution of the responses, and the frequency data were converted to percentages.

#### Results validating the usefulness of the design strategies

We tested the usefulness of the strategies that emerged from the microfluidic patent analysis for generating new ideas for microfluidic devices. Fourteen participants in the idea generation workshop produced 45 (average 3.2) in the first session and 40 (average 2.9) concepts in the second session, without and with design strategies, respectively. As the idea generation sessions were only 20 min in duration, we make no claims about the quantity of concepts generated. Prior work has shown that the use of an ideation tool can increase the time needed to generate a new concept because more cognitive steps are required (Lee et al. 2017).

Our analysis showed that each of the eight design strategies contributed multiple times to concepts generated by participants across numerous projects (Table 2), demonstrating their utility in a variety of microfluidic design contexts.

**Table 2:** The frequency of each design strategy use

Strategy #	Strategy card	Count
12	Change flexibility	5
13	Change geometry	8
14	Change surface interactions	7
16	Contextualize	2
20	Impede	11
27	Mimic natural mechanisms	4
32	Substitute way of achieving function	5
36	Utilize opposite surface	5
	Total	47

**Fig. 5:**
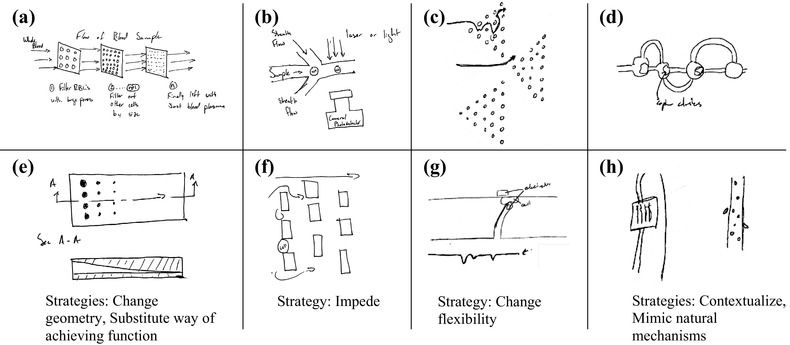
Examples of ideas generated to capture and analyze circulating tumor cells and clusters in blood. Concepts **a**–**d** were generated using participants’ own approaches. Concepts **e**–**h** were generated using design strategies

To protect the identities of participants and their projects, we share example concepts generated for one of the example problems that we provided. The provided problem statement asked participants to design a microfluidic device that could be used to capture and analyze circulating tumor cells (CTC) and clusters in the blood (Appendix 1). A subset of ideas generated during the workshop is shown in Fig. 5. Concepts (a–d) were generated using participants’ own approaches. Concept (a) incorporates layers of porous membranes to sort and capture CTC’s with specific size and deformity. Concept (b) is a microfluidic flow cytometer that can identify cells using a photodetector based on their sizes and morphologies. Concept (c) uses pegs to allow the change of the flow paths of particles based on size and stiffness. Concept (d) is capable of capturing clusters of known size for further analysis.

Concepts (e–h) were generated using design strategies. Concept (e) has a gradient-sloped chamber to sort by size and eliminate the need to have multiple filters, using *change geometry* and *substitute way of achieving function*. Concept (f) incorporates size-dependent single-cell trapping by having a gradient of small gaps along the path, using *impede*. Concept (g), inspired by *change flexibility*, uses a flexible cantilever with electrodes that would deflect as cells passed by. The magnitude of deflection determines cells’ sizes and stiffness levels. Concept (h) is an implantable device with hydrogel channels to allow for CTC invasion and detection, inspired by *contextualize* and *mimic natural mechanisms*. The results show that a subset of design strategies used in this workshop was applicable and helpful in developing diverse concepts.

**Fig. 6:**
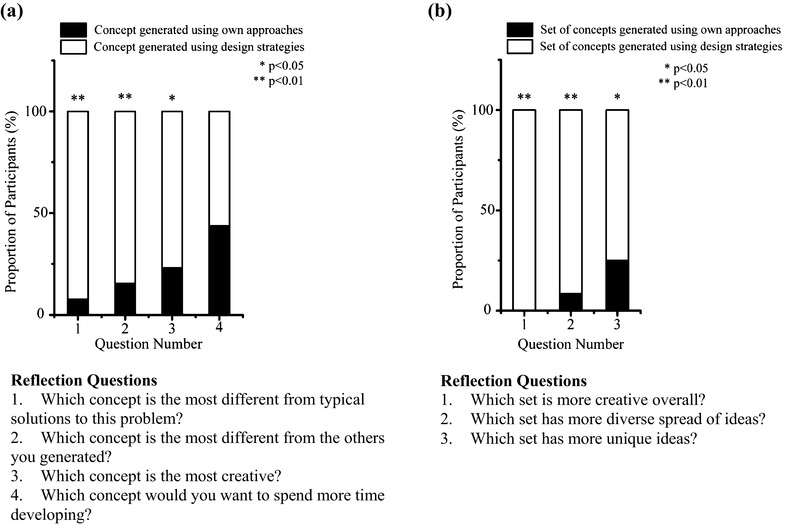
Participants’ reflection data. **a** Participants chose one concept as their most unusual, unique, creative, and suited for development. Concepts generated using design strategies were more often selected as creative and different. **b** Participants compared concepts generated using their own approaches to using design strategies. The sets of ideas generated using strategies were overall more creative, diverse, and unique

Participants evaluated concepts generated using the microfluidic design strategies to most different and creative, with “most different” and “creative” assessed based on participants own expertise in the field (Fig. 6a). When comparing the concepts generated using their own approaches to those generated with design strategies, participants indicated that overall, they viewed the sets of concepts generated using design strategies to be more creative, unique, and diverse (Fig. 6b). These results demonstrate that design strategies helped participants in generating concepts with perceived higher quality.

These results also show that participants did not naturally use these same design strategies when they first generated ideas. Though the strategies may be familiar through knowledge of existing designs and experience, introducing the explicit strategies in the second session resulted in more ideas that were different from ones previously generated, and more creative. This suggests that capturing design knowledge in microfluidics and providing it in the form of a design tool can facilitate idea generation.

## Discussion

The findings from the study demonstrate design strategies evident in microfluidic devices and a method for identifying these strategies. Systematic analysis of microfluidics patents identified 15 strategies that were “inherent” to microfluidics devices, for example, the use of hollow channels, and transparent materials. The analysis resulted in identifying 36 non-inherent strategies that are demonstrated in microfluidic patents. 19 of these were previously identified as part of Design Heuristics (Yilmaz et al. 2016a) and 17 were new strategies only observed in this study. These results suggest that there are common strategies between microfluidic device design and product design, but also domain-specific ones, as well as strategies that are foundation to microfluidics (Squires and Quake 2005).

Our study design was similar to studies that guided the development of TRIZ, the theory of inventive problem solving. TRIZ was produced by rigorously studying strategies of over 40,000 inventions evident in patents, leading to the formulation of 40 principles for finding effective solutions (Altshuller 1997). However, TRIZ principles focus on making tradeoffs and resolving technical conflicts in the later stages of implementing designs. Other studies of product design have used similar methods to identify design strategies from award-winning product designs (Yilmaz et al. 2016b), which led to the identification of Design Heuristics. These heuristics were shown in protocol studies of designers as they created new concepts (Daly et al. 2012c). The 36 non-inherent, novel microfluidic design strategies identified in this study may be useful in helping microfluidic designers introduce more variations in the designs they consider. The best practices of idea generation include coming up with a set of ideas that are different from one another through divergent thinking (Brophy 2001), which leads to novel and innovative ideas to solve a problem (Baer 2014). The microfluidic strategies identified from this study are intended to assist in idea generation in the early stages of a design process, where creative ideas are encouraged before developing them.

Minimal guidance has been available to assist in idea generation in the design of microfluidic devices. The results of the microfluidic strategy tool experimental study demonstrate that microfluidic design strategies can be beneficial for graduate students and postdoctoral researchers, who are practitioners in microfluidic design, to solve important research questions. A variety of design strategies were used both with the same problem and across different problem contexts. This suggests that design strategies are broad enough to suggest concepts but do not determine exact solutions. Furthermore, the use of these strategies as a guide can assist designers in thinking about non-obvious and different solutions to discover creative and diverse concepts, which are best practices in design (Zenios et al. 2009). While microfluidic designers may be familiar with some of the strategies shown from prior experiences, the collection of the strategies assisted in systematically expanding the possibility of concepts during idea generation. While opportunities to support microfluidics in classrooms have been proposed (Fintschenko 2011; Greener et al. 2012; Priye et al. 2012) and microfluidic microfabrication and testing methods to execute design concepts are available (Lee and Sundararajan 2010; Ferry et al. 2011), limited guidance has been provided in the early stages of a microfluidic design process. Using the design strategies can help both novice and practitioners in ideation that can lead to more varied and innovative designs. Assisting microfluidic designers in idea generation to promote original and creative thinking can lead to the development of novel devices that can address important problems in biology and biochemistry.

### Limitations and future work

The set of strategies identified in this paper are based on a sample of 235 patents over a 2-year period. Certainly, other strategies may be evident in a much larger sample. As part of this study, we additionally analyzed 40 randomly selected patents from years 2010–2013, and no new strategies emerged, which suggested saturation. However, additional strategies in microfluidic design may exist, and with advances of technology, more design strategies will likely emerge.

Another limitation is the small sample size for the experimental study with self-selected participants. Thus, the results may not be generalizable across the population of microfluidic designers. While larger studies aim for generalization, our goal was to obtain a detailed understanding of small population to provide a “proof of concept” for future, larger studies. In-depth studies with small sample sizes are common in design research (Goldschmidt 1995; Gosnell and Miller 2015; Cardoso et al. 2016).

Additionally, assessments of the concepts were done by participants themselves. Several potential biases exist in self-assessments. Research in design fixation, an attachment to early solution ideas and concepts (Cross 2001), documented that designers favored their initial concepts despite flaws in those ideas (Ball et al. 1994). Due to fixation, participants may favorably rate their ideas without using design strategies in all aspects including creativity. Reactivity bias may also exist; participants may answer questions based on how they wish to be perceived, and this can have both positive or negative consequences in assessment (Lavrakas 2008). Also, participants may interpret the experiment’s purpose and adapt to the experiment (Rosenthal and Rosnow 2009). Thus, self-assessments may be affected by individual biases but it is not possible to anticipate that biases would favor one direction or another. Self-assessments are an accepted measure of cognitive ability and are commonly used (Shrauger and Osberg 1981; Kreitler and Casakin 2009). We also relied on self-assessments instead of having the research team rate the ideas to eliminate any bias that might be introduced by interpreting participants’ responses. Our study sufficiently demonstrated that the design strategies developed from microfluidic patents had an effect in idea generation and future work could examine the role of biases.

## Conclusion

Innovations can often be traced back to success in idea generation. However, it is difficult to study the processes involved in successful device design in microfluidics. In this study, we utilized patented designs as a sample of successful innovations in devices within the field. We examined evidence in 235 US patents to identify 36 different microfluidic design strategies. In a study with experienced designers, the identified design strategies served as an effective tool to generate more creative and non-typical ideas. Explicit presentation of these design strategies may assist engineers in microfluidics as they generate diverse and creative concepts early in the design process. Furthermore, the use of design strategies can support development of microfluidic devices to provide new insights in biology and biochemistry.

## Appendix 1 Design problem statements given to participants who did not have a problem

### Problem statement 1

Circulating tumor cells (CTCs) are shed from tumors, enter the circulatory system and migrate to other organs to form secondary tumors. Metastasis to distant organs is the leading cause of death in patients with cancer. Numerous studies in the past have shown that CTCs can be potentially used as markers to predict disease progression and survival in metastatic and possibly early-stage cancer patients. As blood collection is simple and minimally invasive, CTCs could be used as a real-time marker for disease progression and survival. Despite this great potential, the use of CTCs faces many hurdles. Biophysical factors that may diminish the detection of CTCs include: (1) filtration of large CTCs in smaller capillaries, (2) clustering of tumor cells that lodge in capillaries, (3) cloaking of CTCs by platelets or coagulation factors, and (4) extremely low concentration in blood. Variations exist in the spatial and temporal distributions of CTCs within the circulation. Indeed, most solid malignancies have typical patterns of metastasis according to the localization of the primary tumor, suggesting at least partial filtration of CTCs. Thus, the site of blood collection for CTC detection may be critical. If CTCs need to be collected from various sites according to cancer type, blood sampling may not be as minimally invasive. Adding to this is the current requirement of large quantities of blood for CTC isolation and detection. Extracting blood from vessels draining the tumor may thus not be feasible and calls for the development of CTC detection devices that can handle small sample volumes. CTCs are found in blood at extremely low concentration, which makes it difficult to isolate and characterize them. A normal concentration in a human cancer patient is approximately 1–100 CTCs per ml of blood.

An ideal CTC capture system would isolate all of the CTCs in the blood sample without accidently isolating other cells and be able to distinctly differentiate and compare heterogeneous CTCs. Design a microfluidic device that can be used to capture and analyze CTCs and CTC clusters in the blood.

### Problem statement 2

DNA is vital for all living beings as it holds the genetic instruction guide for life. In humans, genetic testing can be used to determine a child’s parentage (genetic mother and father) or in general a person’s ancestry or biological relationship between people. In addition to studying chromosomes to the level of individual genes, genetic testing in a broader sense includes biochemical tests for the possible presence of genetic diseases, or mutant forms of genes associated with increased risk of developing genetic disorders. Today, tests involve analyzing multiple genes to determine the risk of developing specific diseases or disorders, with the more common diseases consisting of heart disease and cancer. The results of a genetic test can confirm or rule out a suspected genetic condition or help determine a person’s chance of developing or passing on a genetic disorder. Genetic tests are performed on a sample of blood, hair, skin, amniotic fluid (the fluid that surrounds a fetus during pregnancy), or other tissue.

The current methods of DNA analysis can be costly, time consuming, and not portable. A significant amount of money is allocated towards maintaining the large, sophisticated pieces of technology. Recent years have shown a lot of research focused on reducing the size of the required equipment. Microfluidics presents numerous advantages for DNA analysis by reducing reagent consumptions that lead to cost reduction per analysis and faster analyses due to shorter reaction and separation times. Design a microfluidics device that can be used to analyze DNA.

## Appendix 2 Questions used in the reflection survey

See Tables 3, 4, 5 and 6.

**Table 3:** List of all Design Heuristics (Yilmaz et al. 2016a)

1. Add levels	27. Cover or wrap	53. Reduce material
2. Add motion	28. Create service	54. Repeat
3. Add natural features	29. Create system	55. Repurpose packaging
4. Add to existing product	30. Divide continuous surface	56. Roll
5. Adjust function through movement	31. Elevate or lower	57. Rotate
6. Adjust functions for specific users	32. Expand or collapse	58. Scale up or down
7. Align components around center	33. Expose interior	59. Separate functions
8. Allow user to assemble	34. Extend surface	60. Simplify
9. Allow user to customize	35. Flatten	61. Slide
10. Allow user to reconfigure	36. Fold	62. Stack
11. Allow user to reorient	37. Hollow out	63. Substitute way of achieving function
12. Animate	38. Impose hierarchy on functions	64. Synthesize functions
13. Apply existing mechanism in new way	39. Incorporate environment	65. Telescope
14. Attach independent functional components	40. Incorporate user input	66. Twist
15. Attach product to user	41. Layer	67. Unify
16. Bend	42. Make components attachable or detachable	68. Use common base to hold components
17. Build user community	43. Make multifunctional	69. Use continuous material
18. Change direction of access	44. Make product recyclable	70. Use different energy source
19. Change flexibility	45. Merge surfaces	71. Use human-generated power
20. Change geometry	46. Mimic natural mechanisms	72. Use multiple components for one function
21. Change product lifetime	47. Mirror or array	73. Use packaging as functional component
22. Change surface properties	48. Nest	74. Use repurposed or recyclable materials
23. Compartmentalize	49. Offer optional components	75. Utilize inner space
24. Contextualize	50. Provide sensory feedback	76. Utilize opposite surface
25. Convert 2-D to 3-D	51. Reconfigure	77. Visually distinguish functions
26. Convert for second function	52. Redefine joints	

**Table 4:** New design strategies evident in the study of microfluidic device patents

Microfluidic strategy	Description	Frequency
Add modularity	Design a device comprising of multiple components that can be arranged in multiple layouts and are easily replaced. This can allow for rapid prototyping	2
Add motion	Add motion as part of the device’s function. This can improve function or change user interaction. This applies to the microfluidic device rather than the fluid within it	4
Adjust temperature	Alter the typical or expected temperature of the device’s material or sample	15
Automate	Make the device run with minimal user input. This can improve accuracy of results and usability of the device	8
Change volume–surface area	Highlight areas where the sample interfaces with the device. Altering the volume to surface area ratio can change how much the sample interacts with the surface of the device	2
Create a multi-phase system	Design a device that incorporates multiple phases or immiscible fluids. This can prevent unwanted false positives and cross-contamination/cross-talk	5
Embed electronics or electrodes	Design a device that has built-in electronics or electrodes. This can improve fluid control, increase functionality, and increase usability	23
Impede flow	Explore ways of deviating flow from its preferred path. This can increase interactions between analytes and channel surfaces, and help regulate flow	5
Incorporate filtration, separation, and/or sorting	Explore ways of separating, containing, or isolating parts of samples. This can serve as a method of isolation without knowing biochemical properties. It can also increase sensitivity, decrease contamination, and improve fluid flow	19
Incubate	Store droplets or particles or increase the time it takes for them to move through channels. This can allow for processes that take longer than a few minutes to complete to be incorporated into a microfluidic assay	7
Merge droplets	Consider incorporating droplets that converge. This can control mixing ratios and increase precisions	3
Run on passive flow	Allow the fluid to flow using passive methods, which include the gravitational force and capillary action. This can save energy, eliminate need for external equipment, and simplify the device	4
Run parallel operations	Consider making a device that can run similar operations simultaneously. This further increases throughput, and can decrease run time and cost	24
Simplify sample preparation	Consider treating the sample within the device. This can increase usability by reducing pretreatment steps	9
Use a disposable cartridge	Consider using a small, removable component designed to be inserted into a device. This can reduce cost, increase usability, and increase variety of applications	15
Use colorimetric or fluorescent reading	Utilize visual color changes or fluorescence to detect the status of the samples. This can improve detectability in devices and provides an easy way to visualize the reaction progress	10
Use magnets and/or a magnetic field	Consider incorporating magnets or using a magnetic field for proper device function. This can allow for better control of samples and analytes	12

**Table 5:** The list of transferable design strategies identified from the original Design Heuristics in the microfluidics device patents

Transferable strategy	Description	Frequency
Add to existing product (DH #4)	Use an existing item as part of the product’s function. Consider attaching components or defining relationships between objects. This can reduce material and cost, or improve efficiency	2
Adjust functions for specific users (DH #6)	Design the functions of the device with target user characteristics in mind. This can improve device performance	2
Allow user to customize (DH #9)	Give the user customizable options to best fit their needs and preferences. The final product is developed for each user based on their choices. This can allow the user to use multiple experimental procedures with only one device	13
Apply existing mechanism in new way (DH #13)	Consider whether existing devices or their components can fulfill the desired function. This can facilitate reuse of existing devices, make the design process more efficient, and expand the pool of options	6
Attach independent functional components (DH #14)	Identify different parts or systems with distinct functions, assign form to each, and add a connection between them. This can increase product efficiency, reduce material, facilitate compactness, and unite separate functions	2
Attach product to user (DH #15)	Design the device around the user by attaching it to the user’s body, and redefine how the function is achieved. Consider attachments to a variety of body parts such as the head, finger, back, and feet. This can increase device portability and efficiency	7
Change flexibility (DH #19)	Alter the typical or expected flexibility of the device’s material. This can affect durability, collapsibility, function, and adjustability of the device	13
Change geometry (DH #20)	Alter the typical or expected geometric form of the device or its components while maintaining function. This can redefine user interactions, make the device more intuitive, and suggest new product functions	8
Change Device and/or Sample Lifetime (DH #21)	Consider the assumed lifetime of a device or sample, and alter the number of times it can be used. For example, replace disposable components with reusable ones, or vice versa. This can optimize material use, allow environmentally friendly material use, and decrease waste	5
Change surface interactions (DH #22)	Highlight areas where the sample interfaces with the device by changing hydrophobicity, changing surface tension, or allowing for capture of molecules. This can improve existing function, and improve usability	20
Contextualize (DH #24)	Envision the details of how and where the device will be used, and fit the device to this context. Alternatively, redesign a device to function in a new context. This can specialize the device for target user groups and environments, including point-of-care testing and use in low resource settings	30
Convert 2-D to 3-D (DH #25)	Create a three-dimensional object or environment that improves upon existing 2-D designs. This can provide more accurate modeling environments	4
Incorporate environment (DH #39)	Use the surrounding environment (living or artificial) to perform a part of the product’s function or serve as a product component. This can reduce material, create uniformity with the environment, and increase environmental awareness	2
Incorporate user input (DH #40)	Identify product functions that are adjustable and allow users to make those changes through an interface control, using buttons, sliders, levers, dials, touch screens, etc. This can make the product adjustable to the user’s needs	2
Layer (DH #41)	Build the device through a series of layers of similar or different materials. Different layers can provide a variety of functions	15
Mimic natural mechanisms (DH #46)	Imitate naturally occurring processes, mechanisms, or systems. This can provide efficiency and proven effectiveness	10
Slide (DH #61)	Move one component smoothly along a surface or another component. This can expose or cover surfaces, open or close spaces, and offer options to the user	2
Substitute way of achieving function (DH #63)	Replace one or more components with other designs that can achieve the same function. This can improve device performance, change device cost, and facilitate use of more readily available materials. This may include removing an important or costly piece of equipment, such as a thermal cycler for PCR	10
Utilize opposite surface (DH #76)	Create a distinction between exterior and interior, front and back, or bottom and top. Make use of both surfaces for complimentary or different functions. This can increase efficiency in the use of surfaces and materials, or facilitate a new way to achieve function	2

**Table 6:** The list of inherent strategies from Design Heuristics. The inherent strategies were ones typical in most microfluidic devices due to the constraints of the domain

Inherent strategy	Description
Compartmentalize (DH# 23)	Divide the product into distinct compartments, or add a new compartment. This can separate distinct or complimentary product functions, or create an organizational scheme for multiple functions
Cover or wrap (DH# 27)	Overlay a cover, form a shell, or wrap the surface of the product or its parts with another material
Create system (DH# 29)	Identify the core product functions and define a multi-stage process to achieve the overall goal. Separate the stages to organize functional steps, build a complex function, and increase efficiency
Expose interior (DH# 33)	Reveal the inner components of the product by partially or entirely removing the outer surface, or making it transparent or translucent. This can affect users’ understanding of the product’s function
Hollow out (DH #37)	Identify product volumes and remove a portion of that volume to create a cavity (without impacting structural integrity). This can improve the product’s fit to the user, other products, or its environment
Impose hierarchy on functions (DH #38)	Present the functions of the product in a set order to assist in using the product. Make the steps for each function clear; for example, access to the second function only occurs after the first. This can increase safety, make the product more intuitive, and guide the user through the product’s functions
Mirror or array (DH #47)	Reflect or repeat elements across a central axis or point of symmetry. This can help distribute force, reduce manufacturing cost, and improve esthetics
Reduce material (DH #53)	Remove material from the product by eliminating unnecessary components, reducing volume, or redesigning the product in ways that are more efficient. This can decrease product weight, reduce material cost, allow use in new spaces or with different products, and change esthetics
Repeat (DH #54)	Copy components or products. This can enhance function, allow for multiple simultaneous functions, evenly distribute load, and decrease manufacturing costs
Scale up or down (DH #58)	Increase or decrease any of the physical dimensions of the product or its parts. This can introduce a new function, alter the existing functions, and create options for different users
Separate functions (DH #59)	Identify different functional components of a product and separate them into individual forms. This can change user interaction, make the product more accessible, or allow easier replacement of individual components
Use common base to hold components (DH #68)	Align multiple components on the same base or railing system. This can reduce the number of parts needed, allow users to rearrange components, and make the product more compact
Use continuous material (DH #69)	Identify the different components of the product, and create them out of one continuous material. This can reduce the number of parts or joints, and simplify the product
Use multiple components for one function (DH #72)	Identify primary functions of the product and use multiple distinct components to achieve the same functions. This can maximize functional output
Utilize inner space (DH #75)	Identify inner volumes of the product or create them. Utilize this space to place other product components or a different product. This can increase the compactness and provide storage space
